# Innovative webinar-based training to support evidence-to-recommendation decision-making by national immunization technical advisory groups: multi-country experience with hexavalent vaccines

**DOI:** 10.1016/j.vaccine.2026.128856

**Published:** 2026-08-13

**Authors:** Lisandro Torre, Stephen C. Hadler, Kathryn L. Hopkins, Theresa Sommers, Tyler Best, Sidy Ndiaye

**Affiliations:** a Task Force for Global Health, Decatur, GA, USA; b Sabin Vaccine Institute, Washington, DC, USA; c WHO AFRO, Kintele, Republic of the Congo

**Keywords:** Training methodology, Evidence-to-recommendation training, National immunization technical advisory groups

## Abstract

Since endorsement of the Global Vaccine Action Plan, and continued emphasis under Immunization Agenda 2030, the number of functional National Immunization Technical Advisory Groups (NITAGs) has expanded rapidly, creating increased demand for training in evidence-informed decision making. To support timely national policy decisions relating to immunization, innovative and flexible training approaches are needed. This paper describes and evaluates a novel, webinar-based Evidence-to-Recommendation (EtR) training designed to support NITAGs in developing evidence-based recommendations for transitioning from pentavalent plus inactivated polio vaccine to combined hexavalent vaccines.

Between January and June 2025, a five-part EtR webinar series focused on the hexavalent vaccine transition was delivered to countries, primarily within the WHO African Region, who qualify to receive financial and technical support from Gavi, the Vaccine Alliance, to help introduce new vaccines and strengthen their immunization systems. The series emphasized identification of evidence across EtR domains, and a cohort-based model to facilitate regional peer learning. Effectiveness of the webinar series was assessed through electronic post-webinar surveys and virtual key informant interviews.

NITAGs from 32 countries participated in one or more webinars, including nine in three or more. Across participating countries, participants consistently rated the webinars as useful, particularly valuing country experiences and practical guidance on EtR domains. Qualitative feedback highlighted peer learning as the most valuable component of the webinar series, alongside practice guidance on EtR domains such as feasibility and resource considerations, and NITAGs showed strong interest in continued technical support, practical, peer-to-peer learning or “twinning”, and operational guidance for hexavalent vaccine introduction.

## 1 Background

In 2012, the World Health Assembly endorsed the Global Vaccine Action Plan (GVAP), which stated that by 2020 all countries should create or strengthen National Immunization Technical Advisory Groups (NITAGs) [1]. Since that recommendation was endorsed, the number of countries with functional NITAGs has increased from 41 in 2010 to 170 in 2024, which covers 98% of the global population [2]. This rapid increase in the number of NITAGs led to a large demand for technical training of NITAG members by recognized experts to improve operations, such as developing scopes of work, conflict of interest declarations, meeting planning, and formalizing evidence-based decision-making processes. This emphasis on strengthening NITAG capacity continues to be reflected in the Immunization Agenda 2030 (IA2030) [3].

The role of a functional NITAG is to provide evidence-based technical advice to Ministries of Health on immunization policy [4]. Since the release of the GVAP in 2011, countries have been working with the World Health Organization (WHO), WHO Regional Offices, the U.S. Centers for Disease Control and Prevention (U.S. CDC), and other global partners to establish NITAGs and train them to meet the WHO criteria for functionality [1], [5]. Countries report data on their NITAG functionality annually to the WHO through the WHO/UNICEF Joint Reporting Form (WHO/UNICEF JRF) [6]. NITAGs also evaluate their own maturity using the NITAG Maturity Assessment Tool (NMAT) developed by U.S. CDC, WHO, Global NITAG Network (GNN), and the Task Force for Global Health (TFGH) [7]. The criteria used for assessing the maturity of a NITAG incorporate the seven indicators that assess functionality, including a NITAG’s: 1) Establishment and Composition, 2) Independence and Non-Bias, 3) Resources and Secretariat Support, 4) Operations, 5) Integration into the Policy Making Process, 6) Stakeholder Recommendation, and 7) Ability to Make Recommendations Using Evidence Informed Decision Making Processes [8].

There are several methods for using evidence-informed decision making (EIDM) to guide NITAG recommendations [1], [9]. Since 2017, the TFGH, in collaboration with the WHO, WHO Regional Offices, U.S. CDC, Pan American Health Organization (PAHO), and other global partners, has been training NITAGs on the Evidence-to-Recommendation (EtR) process used by the WHO Strategic Advisory Group of Experts on Immunization (SAGE) and U.S. Advisory Committee for Immunization Practices (ACIP) [10]. In 2021, the TFGH finalized a series of modules into an online training for NITAG members to learn the EtR process. The EtR training includes eight modules designed to be taught by experts with the NITAGs present either online or in-person over three days [11]. Using this approach, TFGH and various partners have trained over 60 NITAGs in the WHO African Regional Office (AFRO), Eastern Mediterranean Regional Office (EMRO), and PAHO on EtR since 2022. These workshops included both individual and multi-country training participation [12].

New priorities from global partners, along with both time and funding constraints, have driven changes in training methodologies for NITAGs in EtR. In 2024, Gavi, the Vaccine Alliance (Gavi) partnered with the Sabin Vaccine Institute (Sabin) to assist countries in switching from the separate pentavalent (penta, DTwP-HepB-Hib) and inactivated poliovirus (IPV) vaccines to a combined hexavalent vaccine (hexa, DTwP-HepB-Hib-IPV). As part of a broader multi-partner effort supported by Gavi, Sabin and collaborators contributed to activities to facilitate country decision-making for the hexavalent vaccine switch, including NITAG capacity strengthening through the EtR webinar series described in this paper. The webinar series was developed in alignment with the timing of the hexavalent vaccine prequalification and the associated Gavi support window, during which many countries were beginning to assess evidence and prepare for NITAG deliberations on the potential switch. This provided an opportunity to re-engage countries in the EtR process with a specific, time-sensitive vaccine policy question.

This switch would reduce the number of recommended DTP-containing vaccine (3 doses) and IPV (2 doses) doses during the first year of life from five to three, increase programmatic efficiency, and enhance protection against poliomyelitis, in line with recent WHO SAGE recommendations on IPV-containing schedules [13]. Ministry of Health requests for NITAG recommendations are a core step in national decision-making to apply to Gavi for co-financing and programmatic support regarding vaccine introductions or antigen switches. Gavi support applications require submission of NITAG recommendations and Inter-Agency Coordinating Committee on Immunization (ICC) approvals.

As a collaborative partner and to support the acceleration of country-level decision-making for the hexa switch, TFGH modified the EtR training methodology by developing an exclusively online webinar series for NITAG members and Expanded Programme on Immunization (EPI) managers to be taught over the course of six months (January–June 2025). This webinar series was open to all Gavi-eligible countries interested in making the switch, though predominantly focused on the African region, in collaboration with WHO AFRO. The webinar series presented the EtR process, focusing on development of a PICO (Population, Intervention, Comparison, Outcomes) question for the hexa switch and defining the evidence needed for each of the seven criteria domains defined by EtR process (GNN training materials), and included suggested sources of information and evidence for each EtR domain. Importantly the webinars included a cohorting component, where countries that had already decided to make the switch (i.e., obtained a NITAG recommendation and/or ICC approval and/or applied to Gavi) shared their decision-making, application, and programmatic action planning experiences and results to their peers.

This webinar series offered new and innovative methodologies in training NITAGs and/or EPI leadership in the EtR process and offered guidance on specific evidence needed to make their recommendation. The webinar series used a cohorting model that allowed countries to share experiences and evidence resources across each EtR domain during the webinars. This paper will describe the new methodologies and explain the benefits and limitations of this training approach.

## 2 Methods

The five-part series of EtR training webinars was completed over six months. The penta to hexa vaccine switch webinars were held approximately every five weeks between January 22 and June 12, 2025. The series targeted NITAG members and EPI managers from Gavi-eligible countries, primarily low- and lower-middle-income countries, interested in applying for financial and technical support from Gavi in switching from penta to hexa vaccine (Table 1).

**Table 1:** Summary of NITAG EtR Webinar series for hexavalent vaccines.

Characteristic	Hexavalent
Number of sessions	5 (Jan–June 2025)
Session length	90 min
Facilitators	TFGH, WHO AFRO^^^
Participants – Country eligibility	WHO AFRO (26) and selected EMRO countries (3)
Prior EtR Training	Many countries (2022–2025)
Participants invited	NITAG chair, members, secretariat
Number participants (range) per session	26–67
No. countries participating in 3 or more webinars	9^⁎^
Homework	Sessions 1-4
Country presentations	1–2 per session
Expert presentations	Each session (see Table 2)
Languages	English & French (all materials)

^Facilitators – TFGH – Hadler, Torre; WHO AFRO – Sidy Ndiaye.

*Countries included Algeria, Benin, Chad, Madagascar, Mauritania, Senegal, South Africa, Uganda, and Zimbabwe.

**Note:** Development of the webinar materials and supporting resources required approximately 80 person-hours, with an additional 10 hours required for webinar delivery and facilitation across the five-part series. These estimates reflect effort by the core training team and exclude participant time.

Each webinar in the series covered one or more specific component(s) of the EtR process, discussed evidence for domain(s) of focus, included country-led presentations, and provided opportunity for peers to ask technical questions and obtain answers (Table 2). During the course of the webinar series, updated WHO SAGE guidance on IPV-containing schedules emerged and was incorporated into the sessions, enabling participants to engage with evolving recommendations in real time. At the end of the series, the participating NITAGs were expected to have reviewed and updated evidence or have made progress toward developing a new evidence-based recommendation for the vaccine PICO question.

**Table 2:** Pentavalent to hexavalent vaccine switch webinar series content.

Webinar Date	Session Topic	Country / Expert Presentations
January 2025	1. GAVI Hexa Switch Program Process 2. Country Presentation – Experience with GAVI switch: Lessons learned 3. Evidence-to-Recommendation process 4. PICO questions for Hexa switch	1. Burundi 2. Madagascar

February 2025	1. Country presentations – PICO question 2. Domain 1 (Problem) 3. Domain 2 (Benefits and Harms)	1. Chad 2. Mauritania 3. Zimbabwe (Experience with considering Hexa switch)

March 2025	1. Country Presentations – Domains 1, 2 2. Domain 4 (Resources) 3. Domain 7 (Feasibility)	1. Mauritania 2. Chad

April 2025	1. Country Presentation – Domain 4 (Resources) 2. Domain 3 (Values and Preferences) 3. Domain 5 (Equity) 4. Domain 6 (Acceptability) 5. Updates on WHO Sage Recommendations and GAVI Support	1. South Africa

June 2025	1. Country presentations – Completing NITAG recommendations for Hexa switch applications 2. Synthesizing Evidence 3. Completing the EtR Framework 4. GAVI update	1. Chad 2. Madagascar

### 2.1 Country participation and language of engagement

The webinar series was conducted primarily in English with French live, simultaneous translation, provided by professional translators with experience in immunization and technical subject matter. The webinar series was initially intended for a closed group of African NITAG members and EPI representatives that were interested in making the vaccine switch. However, participation was opened up to the same stakeholders from all interested, Gavi-eligible African countries and some interested Gavi-eligible countries in other regions (as made aware by Gavi) by the start of the webinar series. Participation of previous webinars was not a hard requirement to attend subsequent webinars, though attending the full series was recommended. NITAG members from most participating countries had been previously introduced to the EtR process through the five-day NITAG workshop series led by WHO AFRO beginning in 2022.

**Fig. 1:**
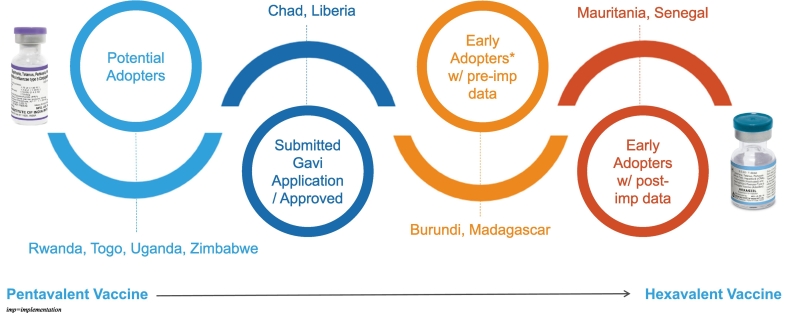
Cohorting of Countries Preparing for GAVI Switch† (from Hexa Early Adopter Workshop Country Switch Phases, as of November 2025). *Early adopter countries are defined as those that had received approval and initiated implementation of the hexavalent vaccine switch at the time of the webinar series. †Remaining country participants were considered ‘interested countries’.

Prior to the start of the webinar series, WHO AFRO, Sabin, and TFGH held an informational webinar for countries that may be interested in the webinar series. The webinar outlined the purpose of the webinar series and expected outcomes. It was followed by a survey to EPI managers and NITAG chairs to assess which countries were considering applying to Gavi for the hexa switch, where were they on the decision-making spectrum, and if they would be interested in joining a regional peer learning webinar series to support their process. Using information gathered through the informational webinar and the online survey, the training team organized countries into four different cohorts, based on expected timeline of NITAG recommendation and subsequent Gavi application submission (Fig. 1). The countries that had already gathered or planned to gather EtR evidence, make NITAG recommendations, and apply to Gavi for support earliest were expected to share their experience with countries with later deadlines.

### 2.2 Webinar series structure

Content of the webinar series is summarized in Table 2. The webinar series introduced homework beginning with webinar 1, which focused on countries identifying their PICO question of interest ahead of webinar 2, and subsequently on finding available country information related to the specific domains discussed in previous webinars (for homework for webinars 2–4). Homework examples from select countries were presented by country representatives (NITAG members and/or EPI managers) at the beginning of the following webinar. The organizers of the webinar series also provided participants an online resource folder (Google Drive), made available throughout the webinar series that contained the presentations in English and French, templates for the work to be completed between webinars, and additional resources such as literature searches and criteria tables.

There were also additional expert presentations, per request of participants or series facilitators, which included: Hexa switch criteria and considerations (Zimbabwe); Hexa switch application experience (Madagascar); Hexa switch cost and cost-effectiveness (South Africa); and Update on SAGE recommendations and Gavi policy on hexa switch application (Gavi) (Table 2). While the EtR framework is standardized and guided the overall structure of the webinar series, participant feedback was incorporated to refine topic selection and country presentations in subsequent sessions. Post-webinar surveys informed the inclusion of expert and country-level examples on topics of interest, such as cost-effectiveness. Webinar sessions were not systematically recorded or disseminated, as the format was designed to prioritize live participation, real-time discussion, and simultaneous interpretation.

### 2.3 Webinar series evaluation

An electronic post-webinar survey and key informant interviews (KIIs) with the participants were used to evaluate engagement and effectiveness of the webinar series. The post-webinar surveys were deployed to all webinar attendees after webinars 2–5. A survey template asked questions regarding what was both helpful and/or missing in the content shared, and their country’s plans for developing a NITAG recommendation. Additional questions sought clarification around country plans for a hexa switch and/or preparing a submission for Gavi support, soliciting needs for technical assistance, and desire for continued ‘NITAG/EPI twinning’ or peer learning around decision-making, Gavi application submission, planning and/or implementation of the hexa switch.

Questions were prepared with a five-point Likert Scale (range 1–5) or as open-ended questions and built into Survey Monkey, one in English and one in French. Following each electronic survey, responses were analyzed in Microsoft Excel for descriptive statistics. Open-ended responses were collated and categorized thematically.

More in-depth feedback on the webinar series was solicited through KIIs using a structured interview guide. Sabin and TFGH conducted a total of six KIIs with a purposively-selected sample of representatives from two different cadres of participant groups (NITAG members and EPI representatives) across five countries. Interviews were supported by live, simultaneous translation in English and French, as needed, and audio-recorded and transcribed after obtaining verbal consent. Interviewers used a tailored interview guide for each cadre of key informant, including 1) participants from “early adopter” countries (Madagascar, Mauritania); and 2) frequent webinar participants (i.e., participants attending at least three of five webinars: Togo, Uganda, Zimbabwe). Interview notes were reviewed by the study team to identify key themes across participants. The qualitative analysis was conducted for programmatic evaluation purposes and did not include formal inter-coder validation.

## 3 Results

The subsections below summarize information about webinar series participation and post-webinar series participation consistency and post-webinar feedback surveys and KIIs.

### 3.1 Participation consistency

After the first webinar, participation in the hexa vaccine switch webinars was opened to all interested countries in AFRO and to selected countries in EMRO (Pakistan, Yemen, Afghanistan). Participant numbers varied from 26–67 across the webinar series, representing 32 countries in total (see Table 3). Participants were from country EPI programs and NITAGs, as well as representative focal points from partner agencies (WHO and UNICEF) and global representatives from Gavi, WHO and U.S. CDC. Across webinars, the majority of participants were from country-level stakeholders (NITAG and EPI), representing approximately two-thirds to three-quarters of attendees per session, with the remainder from global and regional partner organizations (e.g., WHO, UNICEF, Gavi, and CDC). Most countries were represented by 1–3 participants per session, although individual webinars occasionally included a larger delegation (approximately 7–9 participants) from a single country. NITAG representatives usually included the NITAG chair and one or more additional NITAG or Secretariat representatives. NITAG members from nine countries, mainly early adopters or in process of application to GAVI, participated in three or more webinars (Table 1).

**Table 3:** Participation overview.

Webinar	# of Participants	# of Countries	Countries Represented
Webinar 1	26	8	Benin, Burundi, Gabon, Liberia, Madagascar, Mozambique, Uganda, Zimbabwe

Webinar 2	32	10	Afghanistan, Algeria, Chad, Ethiopia, Gabon, Mauritania, Pakistan, Seychelles, Yemen, Zimbabwe

Webinar 3	67	17	Algeria, Benin, Botswana, Burkina Faso, Chad, DRC, Eswatini, Madagascar, Mauritania, Mozambique, Pakistan, Senegal, South Africa, Togo, Yemen, Zambia, Zimbabwe

Webinar 4	32	14	Algeria, Benin, Botswana, Cameroon, Chad, Côte d’Ivoire, DRC, Mauritania, Rwanda, Senegal, South Africa, Togo, Uganda, Zimbabwe

Webinar 5	31	13	Algeria, Angola, Benin, Burkina Faso, Chad, Guinea, Madagascar, Mauritania, Mauritius, Nigeria, Republic of Congo, South Africa, Uganda

TOTAL UNIQUE COUNTRIES		32	Afghanistan, Algeria, Angola, Benin, Botswana, Burkina Faso, Burundi, Cameroon, Chad, Côte d’Ivoire, Democratic Republic of the Congo (DRC), Eswatini, Ethiopia, Gabon, Guinea, Liberia, Madagascar, Mauritania, Mauritius, Mozambique, Nigeria, Pakistan, Republic of the Congo, Rwanda, Senegal, Seychelles, South Africa, Togo, Uganda, Yemen, Zambia, Zimbabwe

### 3.2 Feedback surveys and key informant interviews (KIIs)

A total of 29 participants from the hexa webinars completed the surveys (n=3–10 respondents per webinar (Table 4)). Across the series, participants consistently found the webinars valuable in informing their NITAGs and supporting evidence-based decisions related to a hexavalent vaccine switch. Most respondents rated the sessions as either “Very Useful” or “Somewhat Useful,” with particularly strong positive feedback for Webinar 2 (focus on disease burden, and vaccine safety and immunogenicity) and Webinar 4 (focus on values, acceptability, and equity). A researcher from Sefako Makgatho Health Sciences University in South Africa was invited to share how South Africa evaluated the cost implications (resource use domain) of the switch, which was highly regarded by the participants.

**Table 4:** Survey respondents per webinar

Webinar	Survey Respondents/Total Participants (n/N)	Countries Represented
Webinar 2	8/32	Chad, Mauritania, Yemen, Zimbabwe
Webinar 3	10/67	Algeria, Benin, Eswatini, Madagascar, Mauritania, Pakistan, Togo, Zambia, Zimbabwe
Webinar 4	8/32	Benin, Cameroon, Chad, Mauritania, Togo
Webinar 5	3/31	Algeria, Chad, Uganda

Qualitative feedback emphasized the value of cross-country experience sharing—especially insights from Chad, Mauritania, Zimbabwe, and South Africa—and the practical support that the webinars provided in preparing evidence-based recommendations to Ministries of Health. Respondents specifically highlighted that learning from other countries’ experiences was the most valuable aspect of the webinar. Several participants appreciated how the sessions helped break down specific domains of the EtR framework, such as feasibility and equity, making them more accessible for local policy processes, and noted they provided a helpful refresher from previous EtR and vaccinology trainings.

Participants provided generally positive feedback on the webinar format and delivery, particularly appreciating the inclusion of translation, visual aids, and peer country case studies and presentations. Some suggestions for improvement included distributing translated materials in advance to support engagement, enhancing the quality of French-English interpretation, and making available all resources and materials in both languages. Several participants emphasized a desire for more interaction and discussion. Others recommended simplifying technical content to make it more accessible to diverse stakeholders.

Participants across the webinars expressed a clear interest in continued support and technical assistance to guide their countries through the process of evidence-based decision-making and adoption of the hexavalent vaccine. While the webinar series focused on the hexavalent vaccine transition, participating NITAGs were often engaged in broader immunization prioritization and optimization processes, including those supported by Gavi. As such, the webinars contributed not only to the specific hexa-related decision but also to strengthening general EtR capacity for vaccine policy decision-making. Also noted were the needs for operational guidance, particularly on the steps required to implement the hexa switch, and access to practical tools and templates, such as standardized EtR checklists and recommendation note formats, to support the completion of national recommendations.

KII findings were consistent with those of the post-webinar surveys, specifically citing the most useful part of the webinar series was peer learning from other countries experiences. NITAG interviewees noted desire to engage with and learn from experiences of similar Gavi-eligible countries. Several countries wanted to continue exchanging country level evidence with other participating NITAGs through twinning (e.g., Togo and Chad, Zimbabwe and South Africa, Mauritania and Senegal) or to form consortia of neighboring countries or regions to share experiences and evidence (Uganda). Participants generally expressed strong interest in continued peer-to-peer learning. While participants welcomed continued technical support, most suggestions for future engagement focused on mechanisms to facilitate country-to-country exchange, including twinning arrangements, regional learning networks, and collaboration among countries at different stages of the hexa switch process. All key informants welcomed participating in a workshop for early adopters of the hexa switch, to learn about key operational issues, financial planning, monitoring post-introduction, and guidance on completing the Gavi application and implementation plan.

## 4 Discussion

### 4.1 Effectiveness of the webinar model for EtR capacity strengthening

This webinar series was successful in introducing and/or reinforcing the EtR approach to develop specific vaccine recommendations to multiple interested countries/NITAGs. The webinars communicated information in manageable 90-minute sessions, providing guidance on types and sources of evidence needed, opportunity to identify and curate country-specific evidence between webinars, and to communicate and share information with others participating in the webinar. Participants valued the structured, systematic EtR approach, the use of a PICO question to focus on the vaccine issue, and the structured approach to compiling data. From follow-up surveys and KIIs, almost all respondents were interested in using the EtR approach in future NITAG work. Within months of the webinars, one country NITAG moved forward with a recommendation for the pentavalent to hexavalent switch (Chad).

A key observation was the value and importance of sharing of information among participating countries, through both country and other subject matter expert presentations, and discussion during the webinar. Presentations by Chad, Mauritania, Zimbabwe, and South Africa were highlighted. Country sharing of information was the highest value noted in KIIs; countries desired sharing of information from their regional peer countries. Countries gained practical insights on planning and logistics (feasibility); acceptability; importance of local evidence; resource considerations and cost-benefit examples.

### 4.2 Role of peer learning and cross-country exchange

The webinar series was initially intended to establish twinning between NITAGs specifically interested in the pentavalent to hexavalent vaccine switch. However, both time and financial constraints resulted in changing to the planned webinar series, which was ultimately opened to all interested countries. Overall, this twinning consortium was useful for the hexa switch, providing the opportunity to share experience more widely, albeit less intensively. After completion of the webinars, at least six participating countries expressed interest in moving toward more formalized twinning arrangements, regional learning networks, or forming working groups to continue to sharing evidence, experiences, and implementation lessons.

Following the webinars, most countries reported that their NITAGs were on track to make a recommendation by their chosen Gavi application (tri-annual) deadline with many attributing this progress to the EtR webinar series and associated resources. Respondents cited frequent use of the webinar slides, examples of hexavalent switch criteria tables, the WHO position paper, economic evaluations, and WHO training materials, suggesting the series contributed meaningfully to institutional capacity building.

Nevertheless, countries desired more operational and technical guidance for introduction, resources, checklists, expanding the availability of EtR-related resources in both English and French, and delivering follow-up support focused on high-priority topics such as costing and operational planning. To sustain this momentum, the Sabin-led Hexavalent Vaccine (Switch) Consortium (HeVAC), designed and conducted an in-person Early Adopters Workshop five months post-webinar series (November 2025), to support regional peer learning and provide technical guidance support around previously mentioned high-priority topics. The workshop provided targeted technical assistance to NITAG and EPI representatives from countries considering a hexa switch, with early adopting countries (Mauritania and Senegal) sharing real-world evidence and experience to expand the availability of EtR-related resources in both English and French [14]

### 4.3 Strengths and limitations of the webinar approach

Some important issues were identified during these webinar series. First, country representation was highly variable. Members of many NITAGs only attended a limited number of webinars, and it is uncertain whether/how these participants may have benefitted from the webinar series. One criticism was that the series schedule was not well communicated to all key potential participants. Invitations were sent primarily to the NITAG Chair/Secretariat in AFRO countries, and EMRO countries were not invited to all webinars (as the focused support was for the AFRO region). Suggestions were to make invitations open to all NITAG members across WHO regions, with clear communication of dates and materials disseminated at least three weeks in advance.

Other issues identified in the feedback were that some participants preferred to see more quantitative information (webinars focused on qualitative overview of all parts of EtR); some preferred shorter, less technical webinars and more opportunities for discussion. Although the webinars brought together countries with similar challenges in developing evidence-based recommendations, the webinar format was not as interactive as an in-person training, with fewer questions asked during/after the presentations and limited time for discussion.

We were able to adapt the webinar content and operational logistics based on the real-time post-webinar participant feedback surveys, specifically improving the translation and making translated materials available by the time of subsequent webinars and identifying experts to provide specific information requested by participants. Resources for the webinars were made available on a shared drive for the webinar series (slides, translations, reference materials [e.g., WHO resources, specific papers]); however, few countries had accessed the resource libraries at the time of the KIIs.

Though Chad did make a NITAG recommendation, overall, this webinar series alone was only partially sufficient training to complete an EtR recommendation for several reasons. First, on average, few NITAG members attended (NITAGs can have over ten members), so the EtR approach would have to be more widely communicated to all members and the secretariat—an advantage of in-person EtR workshops with wider and consistent NITAG attendance. Second, countries need sufficient time to gather local evidence required by the EtR PICO specific criteria process. Notably, the webinar series provided an EtR framework and guidance on key resources for hexa vaccines recommendations [15], as well as country examples of these resources.

While only one country progressed to a formal NITAG recommendation within the timeframe of the webinar series (Chad), this likely reflects the time and complexity required for evidence-informed decision-making processes rather than a limitation of the approach. NITAG deliberations often require time to gather and review country-specific evidence, engage stakeholders, and align with national planning timelines. The webinar series supported this process by providing a structured framework, access to relevant evidence, and opportunities for countries to learn from each other’s experiences.

The strong interest in continued twinning and cross-country exchange further highlights the importance of peer learning as an ongoing mechanism for NITAG capacity strengthening. This finding is consistent with broader efforts by the Global NITAG Network and regional partners to promote country-to-country learning and exchange as a complement to formal technical training. Compared to traditional in-person EtR training workshops, which provide intensive but time-limited support, the webinar approach allowed for continued engagement across countries at different stages of the decision-making process, suggesting that it may serve as a complementary approach to existing training models and other peer-learning mechanisms, such as twinning initiatives and early implementer workshops.

### 4.4 Implications for Future NITAG Capacity Strengthening

Several changes in approach could increase the likelihood that a webinar series leads to recommendations for a specific vaccine. While Gavi, Sabin, and TFGH aligned the training series with the timing of the Gavi application for support in early 2025, the series also had to fit within the Gavi-funded hexa switch assessment project timeline. One suggestion would be to use data gathered from newly established WHO vaccine prioritization exercises completed by NITAGs to choose dates for a workshop. With a better understanding of which countries are actively prioritizing the pentavalent to hexavalent switch, it would be easier to combine several countries on the same timeline in a cohort and share resources and establish a realistic timeline of when they would apply for Gavi support. Ultimately, it was decided to permit the more open cohort of countries and the inclusion of countries that were planning on switching products some day in the future.

### 4.5 Limitations

This webinar series had several limitations. First, because of the evolving timeline of the project and the desire to train as many NITAGs interested in the hexa switch as possible, we did not maintain a closed cohort of countries. This limited the interaction between NITAGs and reduced the opportunities for peer-to-peer learning between NITAGs. There was also limited number of responses to the feedback surveys, which may not reflect the full range of country opinions about the webinars.

## 5 Conclusions/takeaway messages

The serial webinar approach is successful to deliver training in the EtR approach for vaccine policy making for a specific vaccine (Box 1). This approach can be most successful when it targets or brings together a group of countries interested in developing policy for the same vaccine and have a similar timeline for recommendations. In addition to the EtR training materials, country sharing of information is considered highly desirable and was a key component of these trainings. Sharing information and resources between countries and NITAGs can lead to ongoing sharing of information and shorten the amount of time needed for a recommendation, and twinning relationships between participating countries can continue after the webinar series ends. These findings reinforce the importance of peer learning, with cross-country exchange serving as a key mechanism through which NITAGs can strengthen decision-making processes. To facilitate success in this type of training, the following recommendations are suggested: 1) Recruit an interested cohort and make webinars open/available to all NITAG and NITAG Secretariat members of those countries; 2) Optimize planning – presentation mode, translation (if necessary), materials for webinar; 3) Make libraries of materials (EtR, criteria tables, key references) easily available for NITAGs; and 4) Allow participation of other countries and partners, as interested.

Box 1 **Key conclusions from webinar series.**

1. The serial webinar approach is successful to deliver training in the EtR approach for vaccine policy making for a specific vaccine.
2. This approach can be most successful when it targets or brings together a group of countries interested in developing policy for the same vaccine.
3. Country sharing of information and peer learning are considered highly desirable and should be a key component of these trainings.
4. To facilitate success in this type of training, the following are suggested:
  a. Recruit an interested cohort, and make webinars open/available to all NITAG and NITAG secretariat members of those countries
  b. Optimize planning – presentation mode, translation (if necessary), materials for webinar
  c. Make libraries of materials (EtR, criteria tables, key references) easily available
  d. Allow participation of other countries/partners, as interested
5. This approach can lead to ongoing sharing of information, and twinning relationships between participating countries

## CRediT authorship contribution statement

**Lisandro Torre:** Writing – review & editing, Writing – original draft, Visualization, Methodology, Formal analysis, Data curation, Conceptualization. **Stephen C. Hadler:** Writing – review & editing, Supervision, Resources, Methodology, Conceptualization. **Kathryn L. Hopkins:** Writing – review & editing, Supervision, Resources, Methodology, Conceptualization. **Theresa Sommers:** Writing – review & editing, Supervision, Resources, Project administration, Methodology, Investigation, Formal analysis, Conceptualization. **Tyler Best:** Writing – review & editing, Project administration, Investigation, Formal analysis, Data curation. **Sidy Ndiaye:** Writing – review & editing, Resources, Project administration, Methodology, Conceptualization.

## Ethics statement

This study was conducted as part of a programmatic evaluation and capacity-building initiative. Based on its design and the nature of participants (professional stakeholders), it was considered exempt from formal institutional ethics review. Participation in interviews and surveys was voluntary, and informed consent was obtained from all participants. No personally identifiable information is included in this manuscript.

All authors attest they meet the ICMJE criteria for authorship.

## Funding

This work was supported by Gavi, the Vaccine Alliance [GAVI100002048].

## Declaration of competing interest

The authors declare that they have no known competing financial interests or personal relationships that could have appeared to influence the work reported in this paper.

## Data Availability

Data will be made available on request.
